# Development of the 10-question household foodwork interactional assessment questionnaire (FIA-Q10)

**DOI:** 10.1186/s12966-024-01671-x

**Published:** 2024-11-28

**Authors:** Leah E. Cahill, Sharon I. Kirkpatrick, Catherine L. Mah, Jennifer LP. Protudjer, Cynthia Kendell, Mary E. Jung, Helen Wong, Ellen T. Crumley, Meghan Day, Karen T. Y. Tang, Yan Huang, Jyoti Sihag, Laura Brady, Karthik K Tennankore, Navdeep Tangri, Rebecca C. Mollard, Dylan MacKay

**Affiliations:** 1 Department of Medicine, Dalhousie University, 5790 University Ave, Room 225, Halifax, NS B3H 1V7 Canada; 2 QEII Health Sciences Centre, Nova Scotia Health, Halifax, Canada; 3 Department of Community Health & Epidemiology, Dalhousie University, Halifax, Canada; 4 School of Public Health Sciences, University of Waterloo, Waterloo, Canada; 5 School of Health Administration, Dalhousie University, Halifax, Canada; 6 Department of Pediatrics and Child Health, University of Manitoba, Winnipeg, Canada; 7 Children’s Hospital Research Institute of Manitoba, Winnipeg, Canada; 8 Institute of Environmental Medicine, Karolinska Institutet, Stockholm, Sweden; 9 School of Health and Exercise Sciences, University of British Columbia, Kelowna, Canada; 10 Faculty of Management, Dalhousie University, Halifax, Canada; 11 Department of Foods and Nutrition, CCS Haryana Agricultural University, Hisar, India; 12 Seven Oaks General Hospital, Winnipeg, Canada; 13 Department of Food and Human Nutritional Sciences, Faculty of Agriculture and Food Sciences, University of Manitoba, Winnipeg, Canada; 14 Chronic Disease Innovation Centre, Seven Oaks General Hospital, Winnipeg, Canada; 15 Internal Medicine Section Endocrinology, University of Manitoba, Winnipeg, Canada

**Keywords:** Household foodwork, Food preparation, Nutrition assessment

## Abstract

**Background:**

Public health nutrition recommendations and clinical dietary interventions emphasize eating healthy food at home, implicitly requiring household foodwork. Household foodwork is defined as the physical and mental tasks a household does for eating meals and snacks. Because no tools exist to measure it, how much time people spend doing household foodwork and the foodwork barriers they experience remain unknown. The objective of the present research was to develop the first stand-alone household foodwork assessment tool.

**Methods:**

Through informal interviews with partners with lived experience, clinicians, and researchers, a literature review, a stakeholder meeting of advisors, and a two-round electronic Delphi process including face/content validation by expert panelists (*n* = 21), we developed the 10-question household foodwork interactional assessment questionnaire (FIA-Q10). An optional accompanying module was developed to collect self-identified demographic data to provide context for understanding how social-structural positionality factors may interact to influence foodwork.

**Results:**

The FIA-Q10 assesses the domains of household composition, frequency of eating at home, special diets within a household, foodwork stress intensity, foodwork barriers, desired supports related to foodwork, and time use for foodwork. The FIA-Q10 measures time use for four subdomains of foodwork among individuals and their households: (1) planning, (2) getting, (3) preparing/cooking, and (4) cleaning up food. In the second Delphi round, the FIA-Q10 scored 95% for language appropriateness, 67% for visual appropriateness, 95% for relevance, 95% for representativeness, and 95% for distribution. Suggested improvements were implemented. All Delphi panelists (100%) reported they would consider using the FIA-Q10.

**Conclusions:**

The FIA-Q10’s development is the first step towards a standardized assessment of foodwork, enabling examination of challenges in foodwork that may impact nutrition and nutrition equity. Future research will focus on FIA-Q10 validation in multiple populations.

## Background

Household foodwork is defined as all of the (usually unpaid) physical and mental tasks required for eating such as: planning, shopping, chopping, cooking, cleaning up, storing, budgeting, monitoring food stock, and accounting for specific needs such as food allergies, special diets, traditions, and celebrations [1–4]. Spending time on food preparation, a component of household foodwork, has been associated with intake of vegetables and fruit, key nutrients, and overall diet quality; [5–7] however, household foodwork otherwise remains mostly unstudied. Public health nutrition recommendations and clinical dietary interventions emphasize eating healthy food at home [8–10], which implicitly requires household foodwork. However, it is unknown which individuals or households participate in foodwork, how much time is spent on household foodwork, and what foodwork-related barriers are experienced.

Recent research has identified this lack of data on household foodwork and called for a standard tool to measure foodwork with feasibility and validity [11–13]. The few studies that have investigated household foodwork-related components (i.e. meal preparation) using single survey questions [14] or time use diaries (which are expensive to conduct and time-consuming to the participant [15–17]) have limitations such as inconsistent coding and including only one individual’s foodwork per household [18–21]. They also often only measure the previous 24-hour period [22], which does not account for many foodwork tasks that do not occur daily, such as grocery shopping. Although barriers to household foodwork may overlap with low levels of food literacy [23, 24], cooking skills confidence [25], and food security [26], tools that measure these related concepts cannot be used to assess household foodwork [1, 25, 27, 28]. An individual with high food literacy or high food security does not necessarily do a lot of foodwork.

There may be many reasons why a person does (or does not do) foodwork, and both low and high amounts of foodwork could have negative health impacts. While food literacy and food security are obvious factors influencing a healthy diet, a 2001 systematic review identified that the most common barriers to dietary adherence among individuals in cardiac rehabilitation programs were time constraints and lack of family support to help obtain or prepare the recommended foods [27]. Indeed, relying on adult children for grocery shopping was a major independent contributor to malnutrition in adults admitted to hospital with clinical malnutrition [28]. Evidence to date suggests that the inequities of foodwork are complex, being influenced by social identities [29]. For example, mothers of children with food allergies (who thus require additional foodwork because they cannot eat many pre-made convenience foods) reported career limitations and worse perceived life status, but these findings were not observed in the corresponding fathers [30]. Because social-structural positionality factors such as age, gender, geographic location, pain, disability, and education level appear to interact to influence foodwork [18, 31–36], foodwork measurement may require knowledge of these positionality variables for accurate contextualization and interpretation.

This paper describes the development of the first stand-alone tool to thoroughly assess household foodwork and the related factors that may interact to influence it, the 10-question Household *F*oodwork *I*nteractional *A*ssessment *Q*uestionnaire (FIA-Q10). We aimed to help enable foodwork measurement, without which incorrect conclusions about the relationship between nutrition and health outcomes could occur in various settings. For example, individuals with chronic disease may appear non-adherent with their prescribed medical diet, or their diet may appear ineffective. However, assessing household foodwork could recognize people that have been unable to do the necessary foodwork for the prescribed diet and relevant supports could be identified. Similarly, foodwork measurement is a direct need of nutrition researchers or program evaluators who require a tool to assess if participants can do (or have done) the necessary foodwork to consume a given nutritional intervention, follow nutrition recommendations, or adequately participate in a program. While it is well-established that the food we eat affects our health [37], many peoples’ food intake does not match the evidence-based recommendations or intervention guidelines from nutrition experts [38, 39]. This large discrepancy between dietary recommendations and food intake may be explained in part if household foodwork is assessed, which may also identify novel targets for interventions to improve nutrition.

## Methods

### Advising team engagement and goal setting

The initial conceptualization of the FIA-Q10 originated during a randomized controlled trial (RCT) of physician-prescribed fruit and vegetable delivery to individuals on medical diets [40]. A tool was needed to assess whether a participant (or someone in their household) could do the required foodwork to eat the study’s intervention food. A literature search revealed that there is no recognized tool to measure foodwork. Thus, we initiated the process of developing a tool [41] by informally interviewing our initial study team of study staff, investigators, clinicians, and patient partners about what they would want in a tool. Clinicians (dietitians and doctors) were also interested in using the tool in clinical settings. The common messages were that researchers, patients, and clinicians wanted a tool to capture the time a person spent on foodwork (alone and in relation to the rest of their household to capture interpersonal dynamics of who does the household foodwork) as well as to capture the specific supports missing and/or barriers to foodwork that people are experiencing. Of the two main time-use measurement methods, stylized estimates, which are an established method used in time use research to ask participants how much time they usually spend on a specific activity during a certain period of time [42] were chosen over the use of a time-use diary to decrease participant burden.

To formalize the tool’s development process and undertake community-engaged translational science, which recommends the engagement of an ‘advising team’ [43, 44] (historically referred to as ‘stakeholders’), our initial study team created and invited a list of potential advising team members including clinicians, hospital administrators, nutritional researchers (clinical trialists, epidemiologists), human ecologists, time use experts, people with lived experience, content experts identified from our literature search, and related organizations and advocacy groups including clinician-specific and disease-specific societies. Seventeen advising team members met once to: (1) develop definitions and identify intended users, (2) discuss goals and guiding principles for the questionnaire, (3) provide input on potential FIA-Q questions, and (4) identify experts to serve as panelists for the Delphi process.

We initially compiled a list of all potentially relevant questions for the FIA-Q identified through their previous use in the literature or suggested by our initial study team. We then identified the repeated or overlapping questions that could be merged, which we presented to the advising team, who agreed. Both the initial study team and the advising team voiced that the resulting list of 14 questions was too long for practical use in most situations and that a shorter version of the FIA-Q should be made to meet the goal of obtaining a snapshot of foodwork within a household (especially time used for foodwork, the interpersonal dynamics of who in the household does foodwork, and foodwork barriers faced) using a maximum of 10 questions (the FIA-Q10). The advising team requested that an optional demographics module accompany the FIA-Q10 to collect self-identified demographic data (i.e. disability, age, geographic location, etc.) if these were not already being collected by a study or clinic to ensure that foodwork data could be analyzed in accurate context.

### Delphi process

We conducted a two-round modified Delphi process to collect expert feedback and identify consensus [45, 46] on the FIA-Q10 content. We did not know ahead of time how many rounds we would need to achieve this and would have done additional rounds if required. An electronic Delphi process was used so that panelists from many different geographic locations could provide anonymous feedback before reading others’ feedback to mitigate the possible power structures and dominance in thought collectives that can occur in a group conversation [45, 46]. Round one of the Delphi process was open from August 16th to September 11th, 2023. 40% of the invited experts participated in round one as panelists (*n* = 27). The Delphi round two invitation was sent only to the 27 panelists who completed round 1 and was open from October 25th to November 15th, 2023. Twenty-one panelists completed round two, which is above the minimum recommended sample size (*n* = 15) [47]. As per established Delphi study principles [45, 46], the anonymous group feedback was shared with panelists between rounds to help ensure that each round evolves in response to earlier feedback and allows panelists to consider the input of others to build consensus. Panelists were sent a report with a pie chart depicting the % agreement for inclusion of questions and a summary of the qualitative feedback provided for each proposed question.

## Delphi process round 1: Foodwork definition and proposed questions for FIA-Q10

Round 1 of the Delphi process presented a proposed definition for the construct of foodwork and a list of proposed questions for the FIA-Q10 and the optional demographics module, and asked the panelists to anonymously provide feedback and vote for the questions they thought should be included. For each question proposed for FIA-Q10 inclusion, panelists were asked “*Should this question be in the FIA-Q10?*” and were given three possible responses: “*Yes*,* as it is*” or “*Yes*,* with revision (changes suggested below)*” or “*No*”. In keeping with recommended Delphi methodology [48, 49], we determined in advance that any proposed question would need an established level of 75% consensus to remain in the FIA-Q10, which is considered to be a high level of consensus [50]. To optimize the questions and definitions of the components of foodwork, panelists were also asked an open-ended question to solicit feedback on the “*wording*,* clarity*,* additional answer options*, etc.” for each proposed question. Additionally, panelists were asked general questions about whether important content was missing, whether all parts of the underlying household foodwork definition/conceptualization were covered, and whether the goal of the questionnaire had been met. All improvements suggested in round 1 were implemented before round 2.

## Delphi process round 2: early face/content validation

Because achieving consensus on which 10 questions to include in the FIA-Q10 only required one Delphi round, round two of our Delphi process aimed to improve the FIA-Q10 using methods recommended for face/content validation of questionnaires for medical research [51, 52] to assess and optimize five domains: (1) visual appropriateness (clear syntax, sufficient white space, and visual accessibility), (2) language appropriateness (clear language and grammar that is easy to follow), (3) relevance (relevant questions are asked), (4) representativeness (response options that represent different people and situations), and (5) distribution (contains response options that could capture a wide range of answers). The panelists were provided a link to the FIA-Q10 and optional demographics module in REDCap and answered questions using a five-point Likert scale ranging from *strongly disagree* to *strongly agree* for each of the five domains. Open-ended questions were also asked to solicit feedback on each specific FIA-Q10 question and for the tool in general so that suggestions for improvement could be implemented. Panelists were also asked general questions about whether important content was missing, and whether the goal of the questionnaire had been met.

## Results

### FIA-Q10 intended users and household foodwork conceptualization

The intended users for the FIA-Q10 were identified as researchers, clinicians, implementation evaluators, and individuals with lived experience (which also includes patients’ family members and the public). Household was defined as “any children, spouses/partners, relatives, and roommates who live with you. A household can also be a person living alone.” The construct of household foodwork was defined as “all the tasks a household does for eating” categorized into four subdomains (referred to as “parts” in the FIA-Q10 tool to simplify language) for estimating time use for foodwork: (1) planning food (2), getting food (3), preparing/cooking food, and (4) cleaning up food, with an accompanying list of examples (Table 1). The conceptualization of these subdomains was advantageous for enabling recall of time use and for examining how household members distribute their time among these four subdomains of foodwork.

**Table 1:** The four subdomains (‘parts’) of household foodwork in the FIA-Q10 for estimating time use

Part	Examples
**Planning food**	Time spent: • planning meals • deciding what to eat and where to get food • seeing what food you have and what you need • planning to minimize food waste • making a grocery list • budgeting for food • comparing food prices • finding coupons • choosing recipes
**Getting food**	Time spent: • going to pick up food such as shopping for food in-person at a store, market, or foodbank (including travel time) • ordering groceries online • putting food away at home • growing, gathering, fishing, or hunting for food for your household to eat This does not include growing food to sell, commercial fishing, and raising animals for meat to sell. This does not include time spent dining in a restaurant or cafeteria.
**Preparing/cooking food**	Time spent: • washing food • chopping, slicing, measuring ingredients • setting the table • serving food • portioning food for later meals • preserving food (e.g., making jam, curing meat) • packing meals for work/school • following a recipe • cooking (e.g., pan-frying, grilling, boiling, putting food in and out of the oven, microwave, air-fryer, etc.). This does not include time spent clearing/cleaning up from preparing/cooking food, or time you cook for paid work outside your home. This is your time that you actively spend cooking, not how long the food cooks for.
**Cleaning up food**	Time spent: • clearing the table • loading/unloading the dishwasher • washing dishes by hand • putting leftovers away • disposing of food-waste This does not include time you clean for paid work outside your home.

FIA-Q10 is the 10-question household foodwork interactional assessment questionnaire

### FIA-Q10 questions selection and consensus

In round one of the Delphi process, only 1 of the 14 potential questions presented to panelists for inclusion in the FIA-Q10 did not reach the agreement threshold required, and it was deleted (Table 2). Multiple panelists independently suggested that another question be merged into a related question, so we combined it with the other question. Multiple panelists independently suggested that a question (‘was last week a typical week for you?’) be addressed in the instructions for the FIA-Q10 instead of asked as a question in the FIA-Q10 and that potential respondents should be instructed about what to do if it was not a typical week. The remaining question, which met the inclusion agreement threshold but was ranked 11th, was omitted.

**Table 2:** FIA-Q10 Delphi round 1 results for question selection and consensus

	FIA-Q10	Demographics
Frequency of question outcome	14 questions	19 questions
Questions omitted because agreement threshold not reached	1 (7%)	2 (11%)
Questions merged into another question	1 (7%)	1 (5%)
Questions moved to be addressed in FIA-Q instructions	1 (7%)	0 (0%)
Questions omitted because ranked < 10th	1 (7%)	0 (0%)
New questions developed	0 (0%)	0 (0%)
Questions (remaining) modified	10 (71%)*	16 (84%)
	**28 experts**	**19 experts**
Do you feel that the questionnaire meets its goal**?	21 (75%)	14 (74%)
Would you consider using the questionnaire?	23 (82%)	16 (84%)

*percent totals reach 100% when not rounded

**The goal of FIA-Q10: to get a snapshot of foodwork (especially time, interpersonal dynamics, barriers) within a household using a maximum of 10 questions. The goal of optional demographics module: to collect self-identified demographic data for clinics and studies that may not already collect it to enable studies of how social-structural positionality factors may interact or intersect to affect household foodwork in 20 or fewer questions so that it does not take more than 20 min for someone to complete

FIA-Q10 is the 10-question household foodwork interactional assessment questionnaire

Regarding the structure of questions, conflicting feedback was received on: (1) the inclusion of “unsure” and “prefer not to answer” options for multiple choice, (2) using a Likert scale versus a number scale, and (3) the inclusion of examples for calculating time used for household foodwork, so we consulted the literature to make evidence-informed decisions about revisions before round two. Although some panelists suggested that FIA-Q10 respondents should not have the option to opt out of a question, after reviewing the literature, we included the “unsure” and “prefer not to answer” options [53, 54]. This allows the distinction of being unsure/uncomfortable [54, 55] and promotes higher response rates by reducing the likelihood of respondents quitting the tool or misreporting to avoid answering a question they did not want to answer [56], without heavily altering the distribution of responses to demographic questions [57]. 

Several panelists questioned our use of 5-point Likert-type scales for the two perception-based questions (amount of food prepared at home and amount of foodwork-related stress) and recommended number scales instead. Therefore, we revised the FIA-Q10 to use number scales from 0 to 10 (an odd-number of responses allows a middle category [58]) with accompanying Likert-style descriptors to benefit from the positives of both discreet and continuous rating scales [59]. Allowing for the possibility to collect numerical data enables a wider range of statistical procedures for these two variables; however, a recent study demonstrated that no psychometric advantages were revealed for response scales beyond 6 options [60], so our future plans include the piloting of both types of scales.

Multiple panellists suggested that we provide examples of calculations for foodwork time use estimates to ease the process for respondents. However, providing examples could limit or influence respondents’ answers, thereby introducing bias. Therefore, we instead provide an optional calculator that tabulates the time a participant enters for different subdomains, thus decreasing the burden of the mathematical calculations while keeping the example-free stylized estimates method commonly used when assessing time use [61, 62].

### FIA-Q10 Face/content validation

In the face content validation, the FIA-Q10 scored 95% agreement (% of panelists who responded ‘agree’ or ‘strongly agree’) for language appropriateness, 67% for visual appropriateness, 95% for relevance, 95% for representativeness, and 95% for distribution (Table 3). Improvements suggested in round 2 did not conflict against each other and were all implemented. 100% of Delphi panelists reported that the order and flow of the FIA-Q10 questions made sense. At the end of round one, 82% of the panelists reported that they would consider using the FIA-Q10 to measure foodwork, and this increased to 100% by the end of round two, although it was a smaller panel for round 2.

**Table 3:** FIA-Q10 Delphi round 2 results: face content validation agreement counts/frequency and precents

	FIA-Q10					Demographics Module				
	Strongly disagree	Disagree	Have no opinion	Agree	Strongly Agree	Strongly disagree	Disagree	Have no opinion	Agree	Strongly Agree
**Visual appropriateness**: the FIA-Q10 uses clear syntax, sufficient white space, and is visually accessible	1 (4.8%)	6 (28.6%)	0 (0%)	10 47.6%	4 (19.0%)	0 (0%)	0 (0%)	0 (0%)	13 (86.7%)	2 (13.3%)
**Language appropriateness**: the FIA-Q10 uses clear language and grammar that is easy to follow	1 (4.8%)	0 (0%)	0 (0%)	13 (61.9%)	7 (33.3%)	0 (0%)	0 (0%)	0 (0%)	8 (50.0%)	8 (50.0%)
**Relevance**: the FIA-Q10 asks questions that are relevant	1 (4.8%)	0 (0%)	0 (0%)	15 (71.4%)	5 (23.8%)	0 (0%)	1 (6.3%)	0 (0%)	8 (50.0%)	7 (43.8%)
**Representativeness**: the FIA-Q10 contains response options that represent different people and situations	1 (4.8%)	0 (0%)	0 (0%)	15 (71.4%)	5 (23.8%)	0 (0%)	1 (6.3%)	0 (0%)	9 (56.3%)	6 (37.5%)
**Distribution**: the FIA-Q10 contains response options that could capture a wide range of answers	1 (4.8%)	0 (0%)	0 (0%)	15 (71.4%)	5 (23.8%)	0 (0%)	0 (0%)	0 (0%)	9 (60.0%)	6 (40.0%)
	No		Maybe	Yes		No		Maybe	Yes	
**The order of questions and flow in the FIA-Q10 make sense**	0 (0%)		0 (0%)	21 (100.0%)		0 (0%)		2 (12.5%)	14 (87.5%)	
The questionnaire meets its goal*	0 (0%)		1 (5.0%)	19 (95.0%)		1 (6.3%)		0 (0%)	15 (93.8%)	
**Would you consider using the questionnaire?**	0 (0%)		0 (0%)	20 (100.0%)		0 (0%)		2 (13.3%)	13 (86.7%)	

*N* = 21 panelists participated in round 2 of the Delphi for both the FIA-Q10 and its accompanying options demographics module

**Goal of FIA-Q10**: to get a snapshot of foodwork (especially time, interpersonal dynamics, barriers) within a household using a maximum of 10 questions

**Goal of optional demographics module**: to collect self-identified demographic data for clinics and studies that may not already collect it to enable studies of how social-structural positionality factors may interact or intersect to affect household foodwork in 20 or fewer questions so that it does not take more than 20 min for someone to complete

### FIA-Q10 domains (‘variables’) for household foodwork

The FIA-Q10 produces variables for the domains of household composition (number and type of household members, including dependents), frequency of eating at home, special diets within a household (number and type), foodwork stress intensity, foodwork barriers (number and type), desired supports related to household foodwork (number and type), and time use (in total and within the individual four subdomains of planning, getting, preparing/cooking, and cleaning up food). For time use, the FIA-Q10 allows for the quantification of time (in minutes or hours) used for foodwork for (a) an individual alone, (b) an individual in ratio to the rest of their household, and (c) the total household, allowing for the examination of interpersonal dynamics of foodwork distribution and within the categories of planning, getting, preparing/cooking, and cleaning up food.

### Demographics module

The optional demographics module includes 17 questions to produce variables for self-identified social-structural positions: age, citizenship status, current gender identity, education level, employment status, rural/urban location, household income, life satisfaction, life stress, marital/relationship status, mental health/ability, physical ability/chronic physical health condition, geographic locations (country and region), race, sex assigned at birth, and sexual orientation. Round one of the Delphi process posed 19 potential questions for the optional demographics module, of which two were deleted, one was merged into another question, and the remaining 16 questions were modified to incorporate panelist suggestions (Table 2). In round two, 100% of panelists agreed that the demographics module was visually appropriate, that language was appropriate, and that the distribution was appropriate. Ninety-five per cent agreed that the demographics questions were relevant and representative (Table 3). A question about sexual orientation was then added. Best practice and language for collecting social-structural demographic data is rapidly evolving, and we will continue to update our demographics module to reflect current practices and language.

### Time frame for foodwork assessment

The decision that the FIA-Q10 would assess foodwork during the previous one-week period (‘last week’ time frame) was based on our advising team’s recommendations and scientific literature [62] and it was not challenged in the Delphi process. The week-long time frame of 7-days was used rather than 24-hours or 3-days because several foodwork tasks, such as grocery shopping, may not occur daily but often do occur weekly [63]. Previous studies have reported that restricting participants to recalling their behavior in the previous week requires less cognitive work than asking participants to ascertain a typical week, which can require substantial cognitive skills [64]. Additionally, typical week questions are more susceptible to both social desirability bias and recall bias [64]. Further, the time frame of the previous week avoids seasonal discrepancies present when asked about ‘typical week’ or ‘last year’.

## Discussion

This paper describes the development of the FIA-Q10, created to fill an acknowledged need to assess household foodwork in clinical and research settings. Our Delphi process confirmed the 10 questions to be included in the FIA-Q10 and enabled us to refine the visual appearance, language, relevance, representativeness, and distribution. To the best of our knowledge, the FIA-Q10 is the first household foodwork assessment tool. The FIA-Q10 and its optional demographics module are available with permission for free use online (by any mobile device or computer) in the REDCap platform and could also be adapted for another platform or for paper printing upon request (for permission and access contact Dr. Leah Cahill at leah.cahill@dal.ca). The questions can be filled out by a respondent alone or with assistance from clinic or study staff. A study/clinic/team may choose to ask only the four questions on time use from the FIA-Q10 if their only interest is foodwork time use estimation. Online, the FIA-Q10 begins with a captcha-style question to prevent bots or artificial intelligence from completing the questionnaire.

### Analytical application

The array of variables (domains and subdomains) for household foodwork produced by the FIA-Q10 can be used as predictor (independent) variables, co-variables (confounders), or outcome (dependent) variables in analyses, as categorical or continuous variables, and can be used alone or together. For example, the FIA-Q10 will provide a team examining foodwork barriers with a yes/no variable for each barrier (was the specific barrier present for a respondent or not) as well as a continuous variable representing the total number of barriers faced for each respondent. As an example of employing two FIA-Q10 variables together in analysis, the variable for level of foodwork stress intensity can be used as an indicator of when a higher amount of time used for foodwork may be a detriment (high foodwork stress) as opposed to a benefit (low foodwork stress), because unlike food security and food literacy where high levels are optimal, foodwork at the highest time-use levels could have suboptimal associations or negative impacts.

The FIA-Q10 measures time use for household foodwork over the course of the last week, differentiating between days of the week, among individuals and their households. These time use data allow study of the interpersonal dynamics of foodwork between household members, while also permitting the calculation of indirect costs of foodwork (time in hours*hourly wage). This costing of foodwork could in turn be added to the out-of-pocket (or direct) costs of a household to calculate total costs of a disease state or the potential cost savings of an intervention which reduces household foodwork.

The FIA-Q10’s optional demographics module uses methods as recommended by and adapted from recognized health research organizations [65, 66] and can be used in situations when self-identified demographics data are not already being collected. The demographics module allows foodwork data to be reported as per recommendations such as the Sex and Gender Equity in Research (SAGER) guidelines [67]. The demographics module can facilitate relevant analysis of how social-structural positions relate to household foodwork, allowing common clinical and public health methods of investigating confounding, stratification, mediation, and interaction [68, 69] as well as the application of the intersectionality analysis framework [70–72], to increase the accuracy of research findings [73], and better examine injustice and inequity in medicine and public health [74, 75]. For example, users of the FIA-Q10 could conduct an applied intersectional analysis to understand how these positionality variables jointly interact to influence household foodwork outcomes such as the total time spent on household foodwork per week, the odds of being the primary person completing the foodwork in a household, and the odds of having a high number of foodwork barriers.

### Limitations

The FIA-Q10 has some limitations. Its current online platform has some visual formatting restrictions that could be improved with the development of an application (‘app’) specifically for the FIA-Q. Secondly, it does not assess multi-tasking activities in its time use assessment which is a common potential source of misclassification bias in time use studies, especially in the domain of unpaid domestic work [76]. For example, people may clean up (e.g., wash a knife) as they prepare/cook (e.g., chop food). Although these activities would not be directly simultaneous, it could be difficult to distinguish time use for them. The FIA-Q10 is currently only available in the English language but will be translated into additional languages upon request. While we took care to ensure that the advising team and Delphi panelists were demographically diverse in geographic location, gender, race, (dis)ability, lived experience, and age as is recommended [77], we did not formally collect these self-identified demographic data for reporting. It also remains unknown how much self-efficacy and self-reflexivity respondents need to complete the FIA-Q10, especially when estimating time use. Estimation of time use may never be exactly accurate to the exact minute or hour, but the FIA-Q10 will distinguish a higher amount of foodwork from a lower amount of foodwork similar to how dietary screeners/questionnaires are commonly used in the field of nutrition to distinguish people who consume a higher amount of a food or nutrient from those who consume a lower amount. Another limitation of the FIA-Q10 is that it has the potential for recall bias, as all memory-reliant tools do [78], especially when estimating time use for other members in the family, which could be either or over- or under-reported. To mitigate recall bias using the established techniques of requiring recall over a short timescale and validating recall against objective measurements of events [78], the FIA-Q10 asks about the previous week [64, 78] and will undergo future validation against a wearable camera and time use diary.

### Future directions

The next step is to further evaluate the FIA-Q10 in multiple populations with validation relative to wearable cameras, time use diaries, and semi-structured interviews. The accuracy and precision of the FIA-Q10’s foodwork time use estimates can be assessed by comparison to time use measured by wearable cameras and time use diaries, and the sensitivity and specificity of the FIA-Q10 to report an absence of household foodwork can be calculated. Validation of the temporal stability and internal consistency should also be assessed along with the construct, convergent, and discriminant validity [25, 79, 80]. While combining the FIA-Q10’s 10 variables into a single scale for household foodwork is not planned, exploratory and/or confirmatory factor analysis may still be conducted for individual foodwork variables created by the FIA-Q10.

### Implications

On an individual level, the FIA-Q10 could be used to identify people with household foodwork challenges along with the specific supports they require to be able to consume their recommended diet. For example, the FIA-Q10 could be used during hospital discharge to plan how a discharged person’s household will be able to complete the required foodwork for recovery, especially if the recovering person is usually responsible for the household’s foodwork. It could be used for a family with a new severe food allergy to identify the gaps in their foodwork plan that they need to fill. Data from the FIA-Q10 could be valuable in monitoring peoples’ progress on medical diets.

On a population level, measuring household foodwork using the FIA-Q10 may bring awareness to common foodwork-related barriers to healthy eating with the long-term goal of identifying targets of intervention and narrowing inequities. Without a foodwork assessment tool, there have been no studies comprehensively reporting the barriers households experience related to foodwork or even the time that households spend on foodwork and how the time is influenced by these barriers. It remains unknown how common and consistent (or erratic) foodwork is within households or how doing foodwork is associated with factors such as food security, diet quality, food purchasing behaviours, nutrition literacy, and health outcomes (i.e. severe allergic reaction incidents, development of complications in diabetes, usage of the healthcare system).

Assessing foodwork using the FIA-Q10 could potentially increase design efficiency of future nutrition studies and elucidate some of the large variation in the relationship between dietary recommendations/instructions and peoples’ actual food intake that troubles the field of nutrition [38, 39]. The original need that inspired the development for the FIA-Q10 was to assess whether participants in nutrition clinical trials of fruit and vegetable delivery could and did do the foodwork required to consume the study’s dietary intervention. However, estimating time used for household foodwork also allows for an assessment of the impact of various types of nutrition-related programs (e.g. did a program teaching food skills or delivering food increase peoples’ efficiency and reduce the time required for foodwork? ), the quantification of unpaid labour costs associated with medical diets, and could also be of importance for other economic analyses such as those evaluating the cost-effectiveness of interventions which directly or indirectly reduce household foodwork, such as a school meal program.

## Conclusions

Although public health nutrition recommendations and clinical dietary interventions emphasize making healthy food at home, it is unknown how much time people use for household foodwork or what foodwork barriers they face. The development of the FIA-Q10 is a step towards providing the tool and context to integrate foodwork into research studies and clinical practice. With nutrition being a top modifiable risk factor for many chronic diseases that are on the rise [81], understanding how the practical factors and inequities of foodwork can hinder people from healthy eating helps inform interventions and policies aimed at supporting healthy and sustainable eating habits.

## Data Availability

Data from the current study are available from the corresponding author upon request. The FIA-Q10 and its optional demographics module are freely available for use upon request, all rights reserved. This questionnaire may not be reproduced, distributed, transmitted, or adapted in any form or by any means without the prior written permission of the copyright holder (contact leah.cahill@dal.ca).

## Abbreviations

FIA-Q10: The 10-question household foodwork interactional assessment questionnaire
REDCap: Research electronic data capture
